# Immunohistochemical Characterization and CT-Derived Volume of Epicardial Adipose Tissue in Patients with Coronary Artery Disease

**DOI:** 10.3390/cells14221760

**Published:** 2025-11-11

**Authors:** Matija Furtula, Igor Zivkovic, Slobodan Micovic, Zoran Tabakovic, Gorica Vidovic, Zelimir Antonic, Jelica Vukmirovic, David Savic, Milovan Bojic, Branko Beleslin, Milan Dobric, Jelena Rakocevic

**Affiliations:** 1 Institute for Cardiovascular Diseases “Dedinje”,11000 Belgrade, Serbia; furtulam1205@gmail.com (M.F.);; 2 Faculty of Medicine, University of Belgrade, 11000 Belgrade, Serbia; 3 Center for Radiology, University Clinical Center of Serbia, 11000 Belgrade, Serbia; 4 Cardiology Clinic, University Clinical Center of Serbia, 11000 Belgrade, Serbia; 5 Institute of Histology and Embryology “Aleksandar Dj. Kostic”, Faculty of Medicine, University of Belgrade, 11000 Belgrade, Serbia

**Keywords:** epicardial adipose tissue, coronary artery disease, immunohistochemistry, cardiac surgery, inflammation, ischemic heart disease

## Abstract

**Highlights:**

**What are the main findings?**

- Patients with coronary artery disease (CAD) showed increased T-cell infiltration and elevated UCP-1 expression in epicardial adipose tissue (EAT).
- EAT volume did not differ between CAD and non-CAD patients but correlated with BMI and was positively associated with UCP-1 and GLP-1R immunopositivity.

**What are the implications of the main findings?**

- The findings suggest that EAT in CAD exhibits a dual profile—both inflammatory and metabolically active.
- The association of EAT volume with UCP-1 and GLP-1R expression highlights the potential immunometabolic role of EAT as a therapeutic target in CAD.

**Abstract:**

Background: Epicardial adipose tissue (EAT) is a visceral fat depot surrounding the myocardium. It contributes to coronary artery disease (CAD) through local inflammation, while its metabolic activity, including the expression of uncoupling protein-1 (UCP-1) and incretin receptors (GLP-1R, GIPR), may exert protective effects. The relationship between EAT immunohistochemical features and imaging-derived volume remains unclear. Methods: We prospectively studied 50 patients undergoing cardiac surgery: 25 with CAD undergoing coronary artery bypass grafting and 25 without CAD undergoing valve replacement. EAT samples were immunohistochemically stained for CD3, CD68, MPO, UCP-1, GLP-1R, and GIPR. Preoperative CT was used to quantify EAT volume. Results: Patients with CAD more frequently had higher CD3 immunopositivity compared to the control group (84.0 vs. 58.3%, *p* = 0.047), with no difference in MPO and CD68 immunoexpression. UCP-1 expression was elevated in CAD patients (*p* = 0.004), whereas GLP-1R and GIPR immunopositivity were similar. EAT volume did not differ between CAD and non-CAD patients (102.87 cm^3^ vs. 99.38 cm^3^, *p* = 0.964) but correlated modestly with BMI (r_s_ = 0.325, *p* = 0.021). UCP-1 and GLP-1R immunopositivity, as well as larger LVEDD (left ventricular end-diastolic diameter), were positively associated with greater EAT volume. Conclusions: EAT in CAD exhibits increased T-cell infiltration and elevated UCP-1 expression, indicating an inflammatory yet metabolically active profile. Larger EAT volume was associated with UCP-1 and GLP-1R expression, underscoring the immunometabolic role of EAT in CAD.

## 1. Introduction

Epicardial adipose tissue (EAT) represents a unique visceral fat depot located in close proximity to the myocardium. Since the recognition that EAT may contribute to the pathogenesis of coronary artery disease (CAD), atrial fibrillation, and heart failure with preserved ejection fraction, there has been growing interest in both experimental and clinical research on this distinct adipose tissue compartment [1,2]. EAT is not separated from the underlying cardiomyocytes or adjacent coronary arteries by a fascial layer, which facilitates their direct bidirectional crosstalk.

Under pathological conditions such as CAD, obesity, and metabolic syndrome, EAT secretes adipokines and pro-inflammatory mediators [3,4,5]. These molecules act locally and may potentially alter the metabolism of both the myocardium and coronary vessels. In parallel, EAT undergoes immune cell infiltration, including macrophages and T lymphocytes, further amplifying local inflammation [6]. In recent years, the connection between EAT and inflammation has become one of the most active research areas about EAT.

Lipotoxity is an additional mechanism through which EAT may contribute to CAD [7]. Under pathological circumstances, epicardial adipocytes may become hypertrophic and change their metabolism. This may lead to uncontrolled release of free fatty acids and accumulation of toxic lipid intermediates, with the potential to cause oxidative stress and damage mitochondria, leading to cardiomyocyte apoptosis.

EAT is considered to be a kind of “brown-fat like” adipose tissue, expressing certain amounts of uncoupling protein-1 (UCP-1) [8]. That way, EAT may exert thermogenic and cardioprotective role. Increased UCP-1 expression in patients with CAD may present the adaptive reaction and protective mechanism against pro-inflammatory milieu in CAD [9]. However, in patients CAD with type 2 diabetes, UPC-1 expression in significantly reduced, indicating less protective role while changing its features into more “white-fat like” adipose tissue [10]. Therefore, “browning” or “whitening” of EAT requires further clarification.

The growing interest in incretin hormone receptors in adipose tissue has prompted investigation of their role in EAT as well. Expression of the glucagon-like peptide-1 receptor (GLP-1R) in EAT is thought to exert a protective effect by reducing local inflammation, enhancing fat utilization, and promoting “browning” of the adipocytes [11]. Another incretin hormone receptor, glucose-dependent insulinotropic polypeptide receptor (GIPR) is also expressed in EAT, mainly in its macrophages, however its role is less certain [12]. Expression of both GLP-1R and GIPR makes EAT not only a passive fat depot but also a potential therapeutic target for incretin-based therapies.

Possibility of CT scan to assess EAT volume and density which can serve as non-invasive markers of its inflammatory and metabolic activity of EAT, as well as a hallmark of higher cardiovascular risk, subclinical atherosclerosis, coronary calcifications, and major adverse cardiovascular outcomes [13,14]. There is no universal definition of normal EAT volume in the general population. Literature data indicate average values ranging from 100 to 125 cm^3^, with higher EAT volume observed in men compared to women, and in patients with CAD compared to non-CAD individuals [15,16,17].

However, immunohistochemical features of EAT and their relation to imaging characteristics of EAT in patients with CAD remain incompletely understood.

Therefore, in this study we aimed to assess the expression of inflammatory and metabolic markers in epicardial adipose tissue of patients with CAD and to explore their association with CT-derived characteristics of EAT. By focusing on the immuno-metabolic properties of EAT, we sought to provide novel insights into its role as a potential mediator and biomarker of coronary atherosclerosis.

## 2. Materials and Methods

This study prospectively included 50 patients admitted to the Institute for Cardiovascular Diseases “Dedinje”, Belgrade, Serbia, for indicated and planned standard cardiac surgical interventions (February 2024 to June 2024). A total of 25 patients with coronary artery disease who underwent surgical coronary revascularization (coronary artery bypass grafting, CABG) constituted the CAD group. The control group consisted of 25 patients without significant coronary artery disease who underwent surgical valve replacement (mitral or aortic). Exclusion criteria were: (1) acute myocardial infarction in the previous month, (2) prior CABG, (3) existing chronic inflammatory disease, (4) current therapy with any GLP-1 receptor agonists, (5) participation in another ongoing clinical trial, and (6) inability to provide informed consent.

### 2.1. Clinical Characteristics and Laboratory Analyses

Demographic data (age, sex), anthropometric parameters (weight, height, body mass index [BMI]), clinical characteristics (cardiovascular risk factors including hypertension, smoking, diabetes mellitus, hyperlipidemia, and family history of cardiovascular disease; comorbidities; and concomitant therapy), as well as laboratory values (leukocyte and erythrocyte counts, hemoglobin, urea, creatinine, glucose, creatine kinase [CK], and B-type natriuretic peptide [BNP]) were collected from patients’ medical records or obtained directly from the patients.

### 2.2. Epicardial Adipose Tissue Sampling and Preparation

During cardiac surgery, a sample of EAT adjacent to the proximal right coronary artery (approximately 1 g) was obtained from each patient as part of the standard surgical procedure. Each EAT sample was immediately fixed in 10% neutral buffered formalin for a minimum of 24 h.

All samples were processed for hematoxylin and eosin (HE) and immunohistochemical staining as previously described [18]. Briefly, the tissue was dehydrated through graded alcohols, cleared in xylene, and embedded in paraffin. Thin sections (4 µm) were then cut and stained with HE or subjected to immunohistochemical analysis.

All EAT samples were coded sequentially (EAT1, EAT2, etc.) based on the order of patient enrollment. The histologists who evaluated the samples were blinded to the patients’ clinical status (presence or absence of CAD).

### 2.3. Histomorphometric Analysis of Epicardial Adipocyte Volume

HE-stained specimens were used to capture digital images at 200× magnification, and the cross-sectional area of 100 adipocytes per EAT sample was measured. Adipocyte volume was then calculated using the following formula [19]:

Radius = √(Area/π)

Sphere volume = (4/3)(π × Radius3)

The average volume of 100 adipocytes was calculated for each EAT sample. Digital images were acquired using a Leica DM400 B LED microscope equipped with a Leica DFC295 digital camera (Leica Microsystems, Wetzlar, Germany) and analyzed with the Leica Application Suite (LAS, v4.4.0) software. Adipocyte cross-sectional areas were measured using the open-source ImageJ software (version 1.54 p).

### 2.4. Immunohistochemical Analysis

For immunohistochemistry, heat-induced antigen retrieval was performed on all EAT samples using citrate buffer (pH 6.0) for 21 min. After each step, sections were washed with phosphate-buffered saline (PBS). A protein block was applied to all samples to reduce non-specific staining. Specimens were then incubated with the primary antibody at the appropriate dilution, with CD3 being the marker of T lymphocytes, CD68 marker of macrophages, and myeloperoxidase (MPO) serving as a marker of neutrophil granulocytes.

Following antibodies were used: Anti-CD3 antibody [SP162] rabbit monoclonal, Abcam ab135372, dilution ratio 1:150; Myeloperoxidase polyclonal antibody, rabbit, Invitrogen PA5-16672, 1:200; Monoclonal mouse anti-human CD68 clone PG-M1, DAKO M0876, dilution ratio 1:100; Anti-UCP1 antibody, rabbit polyclonal, Abcam ab155117, 1:100; Anti-GLP-1R antibody [EPR23507-57] rabbit monoclonal, Abcam ab254352, 1:100; GIPR Polyclonal antibody, Invitrogen PA5-118866, 1:200. Sections were processed using a labeled streptavidin-biotin immunoenzymatic antigen detection system (Mouse and Rabbit Specific HRP-ABC Detection IHC Kit, Abcam, ab93677, Cambridge, UK). Immunoreactivity was visualized with 3,3′-diaminobenzidine (DAB substrate kit, Abcam, ab93677), and sections were counterstained with Mayer’s hematoxylin. Negative controls were included by omitting the primary antibody while following the same protocol.

Immunoexpression for CD3, CD68 and MPO immunopositivity was evaluated semiquantitatively as follows: 0 (no immunopositive cells), 1+ (up to 5 immunopositive cells per HPF), and 2+ (≥5 immunopositive cells per HPF). A total of five fields per section were counted, and the average number of positive cells per HPF is calculated. High power field referred to a 40× objective lens, resulting in 400× total magnification. Crown-like structures (CLS) were defined as dense aggregates of inflammatory immunopositive cells completely surrounding one or more epicardial adipocytes.

Since the aim was to assess UCP-1, GLP-1R, and GIPR immunoexpression specifically in epicardial adipocytes (membranous and/or cytoplasmic) rather than in inflammatory, stromal, or mobile cell populations, a distinct semiquantitative scoring system was applied. The proportion of immunopositive adipocytes per area was evaluated using a 0–3 scale: Expression was scored based on intensity and percentage of positive cells: 0—no staining, 1+—weak staining or <25% positive cells, 2+—moderate staining or 25–50% positive cells, and 3+—strong staining or >50% positive cells.

Two histologists independently evaluated EAT immunoexpression. Inter-rater agreement was assessed with Cohen’s kappa. Calculated kappa coefficient was 0.87, reflecting very good inter-rater agreement. In cases where the two investigators disagreed, a consensus was reached through re-evaluation of the samples.

### 2.5. Ehocardiographic Imaging and CT-Derived EAT Volume

As part of the preoperative clinical assessment, echocardiography and cardiac computed tomography (CT) were performed. These imaging modalities provided measurements of EAT thickness and volume, as well as standard cardiac function parameters, including left ventricular ejection fraction (LVEF), left ventricle end-diastolic diameter (LVEDD) and left ventricle end-systolic diameter (LVESD), and regional contractile function.

All patients underwent cardiac computed tomography (CT) imaging using a 256-slice scanner (Brilliance iCT, Philips Healthcare, Best, The Netherlands), with electrocardiographically (ECG)-gated acquisition applied in all cases.

Depending on the clinical indication, a subset of participants underwent native (non-contrast) cardiac CT scanning, while patients from the control group, scheduled for surgical aortic valve replacement, underwent coronary CT angiography (CCTA) as part of a comprehensive preoperative CT panarteriography protocol [20,21]. During this procedure, patients received an intravenous injection of a non-ionic iodinated contrast agent—iomeprol at a concentration of 400 mg iodine/mL (Iomeron, Bracco, Milan, Italy)—in a volume adjusted according to body weight (ranging from 80 to 115 mL).

Quantification of EAT was performed based on the acquired cardiac CT datasets. All analyses were conducted by a single trained reader (IK) to ensure consistency and minimize inter-observer variability.

Image post-processing was performed using the dedicated medical image analysis software Mimics Medical version 26.0 (Materialise, Leuven, Belgium). For EAT segmentation, a predefined attenuation threshold range of −190 to −30 Hounsfield units (HU) was applied, consistent with established criteria for adipose tissue identification in CT imaging [13,19].

Initial segmentation of EAT was performed automatically, followed by manual correction of contours when necessary to ensure complete inclusion of the pericardial boundaries. Upon completion of segmentation, the software automatically generated the EAT volume data, along with three-dimensional (3D) models of the relevant anatomical structures.

### 2.6. Statistical Analysis

Results are presented as frequency and percentage, mean ± standard deviation, or median with range, as appropriate. Differences between groups were assessed using the independent samples t-test for normally distributed data, and the Mann–Whitney U test for non-normally distributed or ordinal data. Categorical variables were compared using the chi-square test. Correlations between variables were assessed using Spearman’s rank correlation coefficient. Due to the non-normal distribution of EAT volume, quantile regression was performed to evaluate potential clinical predictors of EAT volume using the *quantreg* package in R. A *p*-value < 0.05 was considered statistically significant. All analyses were conducted using IBM SPSS Statistics 22 (IBM Corporation, Armonk, NY, USA) and R software (version 4.3.1; R Core Team, Vienna, Austria).

## 3. Results

**Figure 1:**
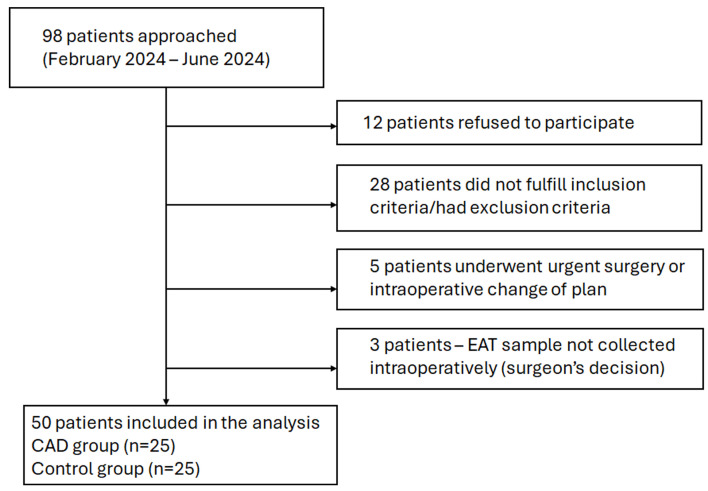
Flowchart of patient screening and inclusion.

During the study period, 98 patients scheduled for cardiac surgery were approached (Figure 1). Twelve patients refused to participate, 28 did not fulfill the eligibility criteria, and in 5 patients urgent surgery or an intraoperative change in the operative plan prevented additional sampling. In 3 patients, an EAT sample was not collected intraoperatively because the operating cardiac surgeon considered that additional sampling would interfere with the planned operative technique. The final study population consisted of 50 patients (25 CAD group/25 controls).

Baseline demographic and clinical characteristics of the included patients are presented in Table 1. In the control group, surgical aortic valve replacement was performed in most patients (*n* = 20; 80%), while surgical mitral valve replacement was performed in 5 patients (20%). The average age of the included patients was 66.5 years, and 80% were male. There were no differences between the groups in the presence of traditional cardiovascular risk factors, except for hyperlipoproteinemia, which was more prevalent in the CAD group (100% vs. 72%, *p* = 0.010). Angina pectoris and previous myocardial infarction were also more frequent in patients with CAD (64% vs. 16%, *p* = 0.001, and 32% vs. 0%, *p* = 0.004, respectively). Statin use was significantly more frequent among patients in the CAD group (88% vs. 52%, *p* = 0.005).

Blood glucose levels were significantly higher in the CAD group compared to controls (mean, 6.71 vs. 5.87 mmol/L, *p* = 0.006). Echocardiographic analysis revealed a lower LVEF in patients with CAD (median 50% vs. 55%, *p* = 0.008). No significant differences were observed between groups in LVEDD or LVESD (*p* = 0.323 and *p* = 0.560, respectively).

### 3.1. Histomorphometric and Immunohistochemical Analysis of EAT

Histologically assessed epicardial adipocyte volume was significantly larger in patients with CAD compared to the control group (mean 0.11 nL vs. 0.08 nL, *p* = 0.008). In the overall cohort, there was no significant correlation between epicardial adipocyte volume and BMI (r_s_ = 0.028, *p* = 0.845), nor was a significant correlation observed within the CAD group (r_s_ = −0.049, *p* = 0.817) or the control group (r_s_ = 0.007, *p* = 0.972).

**Figure 2:**
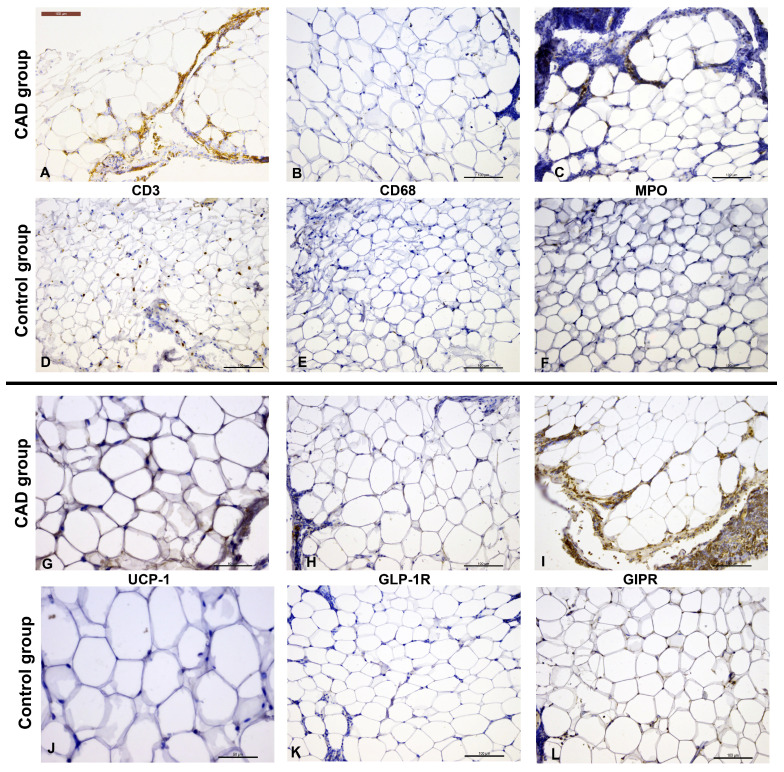
Immunohistochemical staining of EAT from patients with and without CAD (CD31—(**A**,**D**); CD68—(**B**,**E**); MPO—(**C**,**F**); UCP-1—(**G**,**J**); GLP-1R—(**H**,**K**); GIPR—(**I**)—inflammatory cells showing immunopositivity, and (**L**)). Scale bar represents 100 µm, except for UCP-1 images ((**G**,**J**)) where the scale bar represents 50 µm.

CD3^+^ cells were present in 49 patients (98%), where 14 (28.6%) exhibited low CD3 immunopositivity (CD3^+^), while 35 (71.4%) had ≥5 CD3^+^ cells per high-power field (HPF, Figure 2A,D). Patients with CAD more frequently had higher CD3 immunopositivity compared to the control group (84.0 vs. 58.3%, *p* = 0.047).

All EAT samples demonstrated MPO immunopositivity, with 15 patients (30%) showing low MPO^+^ cell extent and 35 patients (70%) showing higher MPO^+^ cell extent. CD68^+^ cells were detected in 42 patients (84%); up to 5 CD68^+^ cells in HPF were observed in 30 patients (71.4%), while 12 patients (28.6%) exhibited higher CD68 immunopositivity. There were no significant differences in the extent of MPO or CD68 immunopositivity between the CAD and control groups (*p* = 0.123 and *p* = 0.769, respectively; Figure 2B,C,E,F).

Crown-like structures (CLS) were more frequently observed in the CAD group compared to patients without CAD (56% vs. 24%, *p* = 0.021).

UCP-1^+^ epicardial adipocytes were present in 32 patients (64%). UCP-1 immunoexpression was significantly higher in patients with CAD compared to the control group (median 2+ vs. 0, *p* = 0.004) (Figure 2G,J). When considering only the presence of UCP-1, without quantification, the difference remained statistically significant (*p* = 0.018).

GLP-1R immunoexpression was detected in 30 patients (60%), while the majority of patients exhibited GIPR^+^ epicardial adipocytes (48 patients; 96%). No significant differences were observed between the CAD and non-CAD patients regarding the presence of GLP-1R or GIPR immunopositivity (*p* = 0.083 and *p* = 1.000, respectively). However, many inflammatory and stromal cells also showed strong GLP-1R and GIPR immunopositivity (Figure 2H,I,K,L).

### 3.2. CT-Derived EAT Volume

**Figure 3:**
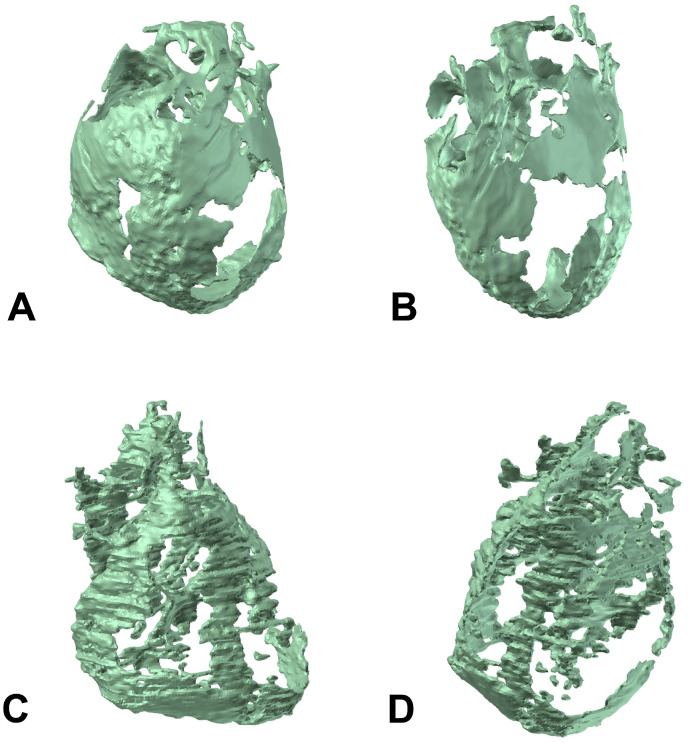
Three-dimensional model generated by the EAT volume data of a patient with higher EAT volume (**A**,**B**), and patient with lower EAT volume (**C**,**D**).

Average CT-derived EAT volume showed no difference between patients with and without CAD (median, 102.87 cm^3^ vs. 99.38 cm^3^, respectively, *p* = 0.946, 95% CI for the median difference −26.9 to 20.0, Figure 3). EAT volume was significantly larger in man compared to women (median, 109.10 cm^3^ vs. 87.02 cm^3^, respectively, *p* = 0.049, 95% CI for the median difference −40.0 to −0.1) There was small, yet significant positive correlation between EAT volume and BMI, with higher BMI values being associated with larger EAT volume (r_s_ = 0.325, *p* = 0.021, 95% CI 0.017 to 0.570).

Quantile regression analysis showed that at the 75th percentile, BMI was positively associated with epicardial adipocyte volume, with each unit increase in BMI corresponding to a 4.9 cm^3^ higher EAT volume (β = 4.9, 95% CI, 0.56–7.88, *p* < 0.05). (Table 2). Additionally, LVEDD was positively associated with EAT volume in all quantiles analyzed (25th, 50th, and 75th percentiles; β = 2.18, 2.81, and 4.02, respectively; all *p* < 0.05), suggesting a uniform effect of LVEDD across the EAT volume distribution.

**Figure 4:**
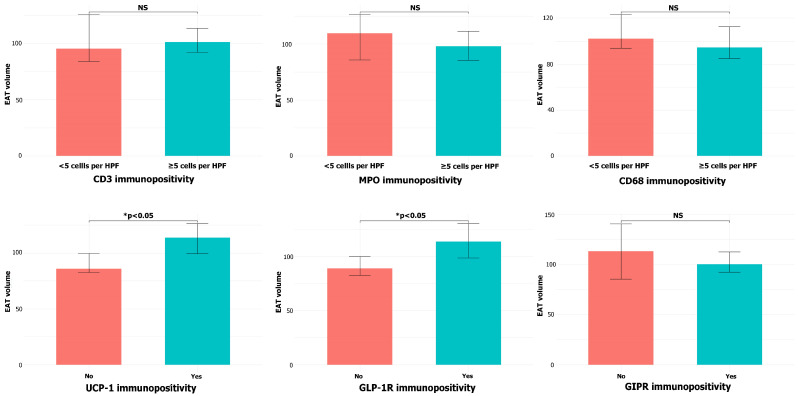
EAT volume depending on CD3, MPO, CD68, UCP-1, GLP-1, and GIPR immunopositivity in the samples of EAT (NS = non-significant).

Higher EAT volume was associated with the presence of UCP-1^+^ cells (median 110.50 cm^3^ vs. 88.82 cm^3^, *p* = 0.010) and GLP-1R immunopositivity (median 115.06 cm^3^ vs. 91.32 cm^3^, *p* = 0.022, 95% CI for the median difference −45.69 to −7.16, Figure 4). There was no difference in EAT volume in patients with different CD3, MPO, or CD68 immunopositivity extent (*p* = 0.439, *p* = 0.775, and *p* = 0.301, respectively), nor with the presence of GIPR^+^ cells (*p* = 0.690).

Subgroup analysis confirmed the association between higher EAT volume and the presence of UCP-1^+^ and GLP-1R^+^ cells in CAD patients (*p* = 0.049 and *p* = 0.018, respectively), but not in the control group (*p* = 0.064 and *p* = 0.320, respectively).

## 4. Discussion

In this study, we found increased T-cell infiltration in the EAT of patients with CAD compared to non-CAD individuals, accompanied by enhanced immunoexpression of UCP-1 in epicardial adipocytes. Moreover, larger CT-derived EAT volume was associated with UCP-1 and GLP-1R immunopositivity, a relationship observed in patients with CAD but not in the control group. These findings indicate significant local inflammatory and metabolic alterations of EAT in the setting of CAD, which may influence disease progression and prognosis. Additionally, LVEDD was positively associated with EAT volume across all analyzed quantiles, as well as with the highest BMI quartile.

Our study demonstrated that epicardial adipocytes were significantly larger in patients with CAD undergoing CABG compared with controls, consistent with previously published studies [22,23]. However, epicardial adipocyte volume did not correlate with BMI, suggesting that local factors within EAT, rather than systemic obesity, may play a more decisive role in its remodeling. It is well established that EAT secretes pro-inflammatory cytokines (such as IL-1β, IL-6, and TNF-α) and pro-fibrotic adipokines (such as TGF-β), which exert paracrine effects on adjacent cardiomyocytes, promoting oxidative stress, endothelial dysfunction, and fibrosis [24]. Recently, a novel adipokine hypothesis was proposed to explain the pathogenesis of heart failure with preserved ejection fraction (HFpEF) [25]. This concept emphasizes the expansion and transformation of visceral adipose tissue, including EAT, leading to systemic inflammation, plasma volume expansion, cardiac hypertrophy, and fibrosis. Our results support the proposed hypothesis with one of the key findings. Namely, positive association between LVEDD and EAT volume was observed consistently across all quantiles, suggesting that larger LVEDD values are linked to greater EAT accumulation. That way, EAT enlargement may be implicated in the pathogenesis of LV enlargement.

Immunohistochemical analysis in our study revealed a pronounced inflammatory profile of EAT in patients with CAD. CD3^+^ T lymphocytes were almost universally present, and higher CD3 immunopositivity was significantly more frequent in the CABG group, supporting the concept that adipose tissue inflammation contributes to the pathogenesis of CAD and atherosclerotic progression [6,26]. Multiple studies using flow cytometry, ELISA, and immunohistochemistry have consistently demonstrated that both the proportion and infiltration of CD3^+^ T lymphocytes in EAT are significantly higher in patients with CAD than in controls [27,28]. This supports the hypothesis that adaptive immunity contributes substantially to the inflammatory milieu of EAT. T and B lymphocytes are known to recruit macrophages and other effector cells, thereby amplifying local inflammation and sustaining a pro-atherogenic environment [27]. Consistently, increased macrophage infiltration in EAT of CAD patients has been documented [28,29], suggesting a crosstalk between adaptive and innate immunity that drives tissue remodeling and may directly influence coronary pathology. However, our results did not show difference in the presence of CD68^+^ macrophages in EAT of patients with or without CAD. Additionally, EAT samples from patients with CAD in our study showed no difference in the presence of neutrophil granulocytes (MPO^+^ cells) compared with patients without CAD, while literature data on neutrophil presence in EAT remain scarce. Importantly, crown-like structures (CLS), histological markers of dysfunctional adipose tissue, were more prevalent in the CABG group, indicating enhanced adipocyte apoptosis and macrophage infiltration.

Another key finding was the increased expression of UCP-1 in epicardial adipocytes of patients with CAD. While UCP-1 is classically regarded as a marker of “browning” and adaptive thermogenesis with potentially protective effects, its higher expression in this setting may reflect a compensatory response to inflammatory stress and oxidative damage. Several studies have shown that EAT may exhibit brown fat-like properties due to higher UCP-1 expression level compared to other fat depos [8,9,30]. Direct comparisons of UCP-1 expression between CAD and non-CAD patients have yielded conflicting findings. Some studies reported increased UCP-1 mRNA and protein levels in the EAT of CAD patients compared with controls, suggesting an adaptive, potentially protective response to cardiac injury [9]. In contrast, other investigations—particularly those including patients with type 2 diabetes—have demonstrated reduced UCP-1 expression, pointing to a loss of brown-like adipose characteristics and a possible adverse impact on metabolism and disease progression [10]. Our results have demonstrated more prevalent UCP-1 immunoexpressing among patients with CAD compared to the control group, which was further supported with the association of larger EAT volume with UCP-1 immunoexpression. Furthermore, GLP-1R and GIPR were detected in a majority of samples, but without differences between CAD and control groups. The strong immunopositivity of inflammatory and stromal cells for these receptors suggests complex paracrine interactions between adipocytes, immune cells, and the adjacent myocardium and coronary arteries.

Previous studies have consistently reported significantly higher EAT volume in CAD patients, with larger EAT volume correlating with CAD severity [22,31,32]. In a meta-analysis including 21 studies and nearly 5000 patients, Wang et al. [20] demonstrated that both EAT volume (assessed by CT) and EAT thickness (assessed echocardiographically) were significantly greater in patients with CAD compared to controls (*p* < 0.001). In contrast, our results did not show a difference in CT-derived EAT volume between groups, which may be explained by the relatively small sample size of our study. A similar finding was observed in the smaller study by Jolfayi et al. [33], which included 21 CAD patients and 20 non-CAD individuals. Nevertheless, a recent meta-analysis including nearly 20,000 patients confirmed that increased EAT thickness and volume have prognostic value in predicting major adverse cardiovascular events [34].

Our findings highlight a notable link between EAT volume and its metabolic and immunohistochemical profile. Higher EAT volume was associated with the presence of UCP-1^+^ and GLP-1R^+^ cells, suggesting that larger EAT depots exhibit more pronounced metabolic activity. UCP-1 is traditionally considered a marker of thermogenic activity, while GLP-1R expression reflects incretin-related signaling; both may represent compensatory or adaptive mechanisms within EAT in response to local stress [12,35]. Increased UCP-1 expression in epicardial adipocytes of CAD patients is generally considered a compensatory/adaptive response aimed at enhancing local energy dissipation and mitigating inflammation. However, under conditions of chronic or severe disease, excessive UCP-1 activity might become maladaptive, potentially contributing to increased lipid mobilization and oxidative stress. Interestingly, no associations were observed between EAT volume and markers of inflammation such as CD3, MPO, or CD68, nor with GIPR immunopositivity, indicating that inflammatory cell infiltration and incretin receptor distribution may be largely independent of adipose tissue volume.

The observed correlation of EAT volume with metabolic markers (UCP-1, GLP-1R) but not with inflammatory markers (CD3, CD68, MPO) highlights the complex biology of epicardial adipose tissue. One possible explanation is that EAT volume primarily reflects the amount of lipid-rich, metabolically active adipocytes, which are closely linked to metabolic signaling pathways. In contrast, inflammatory cell infiltration may be spatially heterogeneous and influenced by local microenvironmental factors, such as proximity to coronary plaques, cytokine gradients, or vascular supply, and thus may not scale directly with tissue volume. These findings suggest that volumetric measures of EAT capture its metabolic activity more reliably than its inflammatory status and underline the importance of combining quantitative and qualitative assessments to fully characterize EAT in the context of coronary artery disease.

Furthermore, these findings suggest potential therapeutic avenues, as modulation of metabolic pathways—via GLP-1 receptor agonists, dual incretin receptor agonists (twincretins), or anti-inflammatory interventions—may alter EAT composition and function, leading to improved metabolic efficiency, attenuation of adipose inflammation, and favorable cardiovascular effects.

Subgroup analyses revealed that the associations between EAT volume and both UCP-1 and GLP-1R positivity were present only in patients with CAD and absent in controls. This observation may suggest that in the context of coronary atherosclerosis, expansion of EAT is coupled with an adaptive upregulation of metabolic activity, possibly as a response to heightened inflammatory or ischemic stress. Conversely, in individuals without CAD, EAT expansion may reflect more benign fat accumulation without significant changes in metabolic phenotype.

Our study has several limitations, including a relatively small sample size and the semi-quantitative nature of immunohistochemical assessment. Moreover, functional analyses such as cytokine profiling or receptor signaling activity were not performed, which limits the interpretation of the biological impact of these findings.

Recent studies have suggested that not only the amount, but also the composition and density of EAT may have important pathophysiological implications [36]. Pericoronary EAT density, as assessed by the fat attenuation index (FAI), is considered to reflect the metabolic and inflammatory activity of the adipose tissue. When adipose tissue becomes inflamed or metabolically active, it undergoes lipid depletion and an increase in water content, leading to higher CT attenuation values. Therefore, a higher FAI indicates greater inflammation and metabolic activity of the adipose tissue, potentially providing additional information beyond EAT volume alone. This concept supports the notion that qualitative changes in EAT may be more relevant for cardiovascular risk assessment than quantitative measures such as total EAT volume. However, in our study, FAI measurements could not be performed, as software capable of such detailed analysis was not available. These measurements would be necessary to assess inflammatory parameters within EAT, since FAI may serve as an important marker of pericoronary adipose tissue inflammation. This limitation could serve as a basis for future research.

However, our interdisciplinary approach combining clinical, radiological, and immunohistochemical characteristics highlight a potential protective metabolic adaptation in CAD and suggest that EAT may serve as both a biomarker and a therapeutic target, particularly for incretin-based strategies [37]. Although previous EAT imaging studies primarily focused on volumetric assessment, our work combines quantitative CT-derived EAT analysis with immunohistochemical evaluation of key metabolic and inflammatory markers. This integrative approach allows us to link EAT volume with local metabolic activity, as reflected by UCP-1 and GLP-1R expression, providing mechanistic insight into how epicardial adipose tissue may contribute to CAD. By demonstrating that EAT volume correlates with metabolic, but not inflammatory markers, our study highlights the functional heterogeneity of EAT and suggests that volumetric imaging alone may not fully capture its biological activity. These findings emphasize the importance of combining imaging and molecular analyses to better understand the pathophysiological role of EAT. Future studies should clarify whether UCP-1 expression and incretin receptor activity in EAT exert adaptive or maladaptive effects in the context of CAD.

## 5. Conclusions

Results of our study indicate that EAT volume per se does not discriminate patients with CAD from controls, but its biological activity, reflected by UCP-1 and GLP-1R expression, may provide insights into disease-specific remodeling of this visceral fat depot. Future studies should address whether these metabolic markers represent protective or maladaptive responses and whether they can serve as targets for therapeutic modulation of EAT in CAD.

## Figures and Tables

**Table 1:** Demographic, clinical, laboratory and echocardiographic characteristics of the included patients.

Characteristic	CAD Group (n = 25)	Control Group (n = 25)	*p* Value
Male, n	22 (88%)	18 (72%)	0.157
Age (years) *	64 (50–75)	69 (43–78)	0.759
BMI (kg/m^2^) **	29.4 ± 4.7	28.4 ± 4.4	0.936
NYHA			
I	6 (24%)	5 (20%)	
II	14 (56%)	14 (56%)	0.913
III	5 (20%)	6 (24%)	
HTA, n	25 (100%)	23 (92%)	0.490
HLP, n	25 (100%)	18 (72%)	0.010
Diabetes, n	9 (36%)	8 (32%)	0.765
Family history of CVD, n	19 (76%)	15 (60%)	0.225
Smoking, n	17 (68%)	13 (52%)	0.248
Angina pectoris, n	16 (64%)	4 (16%)	0.001
Previous MI, n	8 (32%)	0	0.004
Previous PCI, n	0	1 (4%)	1.000
Previous stroke, n	1 (4%)	1 (4%)	1.000
RAAS inhibitors, n	18 (72%)	23 (92%)	0.066
Statins, n	13 (52%)	22 (88%)	0.0005
Leukocytes (×10^9^/L) *	7.3 (5.2–13.6)	7.2 (3.8–11.7)	0.346
Erythrocytes (×10^12^/L) **	4.66 ± 0.43	4.60 ± 0.60	0.664
Hemoglobin (g/L) *	143 (115–156)	140 (101–177)	0.763
Urea (µmol/L) *	6.50 (3.13–15.81)	6.67 (4.49–17.87)	0.839
Creatinine (µmol/L) *	89.4 (60.9–176.0)	85.3 (46.0–125.9)	0.260
Glucose (mmol/L) *	6.71 (5.38–12.34)	5.87 (5.26–11.63)	0.006
CK (U/L) *	95 (12–264)	91 (51–529)	0.930
LVEF (%) *	50 (30–60)	55 (30–65)	0.008
LVEDD (mm) **	52.5 ± 5.3	53.4 ± 7.3	0.323
LVESD (mm) **	34.0 ± 5.1	33.2 ± 7.3	0.560

* Median (range); ** mean ± standard deviation; CAD—coronary artery disease; NYHA—New York Heart Association classification (according to symptom severity); HTA—hypertension; HLP—hyperlipoproteinemia; CVD—cardiovascular disease; MI—myocardial infarction; PCI—percutaneous coronary intervention; RAAS—renin–angiotensin–aldosterone inhibitors; CK—creatin kinase; LVEF—left ventricle ejection fraction; LVEDD—left ventricular end-diastolic diameter; LVESD—left ventricular end-systolic diameter.

**Table 2:** Quantile regression analyses for clinical predictors of epicardial adipose tissue volume across selected quantiles.

Variable	β (25th Quantile)	95% CI	β (Median)	95% CI	β (75th Quantile)	95% CI
Age (years)	0.08	−0.61; 1.07	0.92	−0.20; 1.78	1.12	−1.03; 2.08
Gender	9.58	1.95; 23.99	16.72	2.90; 36.35	38.02	−8.63; 51.74
BMI (kg/m^2^)	2.96	−0.45; 4.34	2.55	0.82; 5.42	4.90 *	0.56; 7.88
CAD	0.47	−13.83; 12.12	3.50	−9.93; 19.35	7.81	−59.55; 26.76
LVEF (%)	−0.84 *	−1.38; 0.18	−0.60	−1.71; 0.20	0.69	−2.61; 1.45
LVEDD (mm)	2.18 *	0.29; 4.38	2.81 *	0.78; 4.45	4.02 *	0.93; 4.04
LVESD (mm)	1.37	0.59; 2.65	1.21	0.75; 3.15	3.40	−0.31; 6.57
CK (U/L)	−0.08	−0.24; 0.02	−0.02	0.22; 0.08	−0.15	−0.42; 0.12
Glucose (mmol/L)	−1.20	−2.30; 3.57	1.63	−2.64; 6.73	−4.88	−9.24; 15.88
Urea (µmol/L)	−0.78	−2.37; 3.19	1.19	−3.71; 2.72	−1.73	−2.37; 1.01
Creatinine (µmol/L)	0.05	−0.27; 0.43	0.23	0.04; 0.39	0.10	−0.18; 0.91

BMI—Body mass index; CAD—coronary artery disease; LVEF—left ventricle ejection fraction; LVEDD—left ventricle end-diastolic diameter; LVESD—left ventricle end-systolic diameter; * *p* < 0.05.

## Data Availability

Created new data is not available due to privacy restrictions.

## Abbreviations

The following abbreviations are used in this manuscript:

CAD	Coronary artery disease
CABG	Coronary artery bypass grafting
CLS	Crown-like structure
CVD	Cardiovascular disease
EAT	Epicardial adipose tissue
GIPR	Glucose-dependent insulinotropic polypeptide receptor
GLP1-R	Glucagon-like peptide receptor
HLP	Hyperlipoproteinemia
HTA	Hypertension
LVEDD	Left ventricular end-diastolic diameter
LVEF	Left ventricle ejection fraction
LVESD	Left ventricular end-systolic diameter
MI	Myocardial infarction
MPO	Myeloperoxidase
PCI	Percutaneous coronary intervention
NYHA	New York Heart Association classification
UCP-1	Uncoupling protein-1
